# ASSESSMENT OF CAREGIVER REPORTED VISIT-ASSOCIATED LOGISTICAL CHALLENGES FOR CLINICAL VISIT COORDINATION

**DOI:** 10.1093/geroni/igae098.3125

**Published:** 2024-12-31

**Authors:** Victoria Ngo, Elizabeth Marfeo, Steven Shirk, Elizabeth Chamberlin, Lauren Moo

**Affiliations:** Department of Veterans Affairs, Bedford, Massachusetts, United States; Tufts University, Medford, Massachusetts, United States; Department of Veterans Affairs, Bedford, Massachusetts, United States; Department of Veterans Affairs, Bedford, Massachusetts, United States; Department of Veterans Affairs, Bedford, Massachusetts, United States

## Abstract

Family caregivers (FCG) of persons living with dementia face high care burden as the care recipient ages, especially with care coordination of in-person healthcare visits. As FCG burden increases, there is an increased likelihood of missed appointments and negative health outcomes for both the FCG and the care recipient. Limited evidence exists describing the impact of managing complex visit-associated logistical challenges FCGs face when coordinating appointments. To address this gap, this study uses a care coordination framework to develop a new assessment—the Visit-Associated Logistics Assessment (VALA). We adopted a three-phased approach: (1) multidisciplinary expert feedback to establish content coverage; (2) cognitive interviews to refine item wording; and (3) field testing to establish the feasibility and usability of the VALA among a clinical sample of FCG of persons living with dementia (n = 20). Results from the first two development phases yielded six functional domains: (1) scheduling, (2) visit preparation, (3) time/travel, (4) occupational impact, (5) routine disruption, and (6) psychosocial well-being. Initial end-user feasibility was established. The VALA had a low respondent burden, shown by minimal missing data, and appropriate content coverage, as indicated by an acceptable distribution of responses across items and response categories. The VALA represents the first FCG-focused assessment characterizing logistical factors for helping older persons attend medical visits. Initial phases of content validation and usability testing support the use the VALA within clinical and community-based settings to better support management and care coordination of in-person clinical visits for FCGs of persons living with dementia.

